# Clinical practice guideline for transurethral plasmakinetic resection of prostate for benign prostatic hyperplasia (2021 Edition)

**DOI:** 10.1186/s40779-022-00371-6

**Published:** 2022-04-01

**Authors:** Xian-Tao Zeng, Ying-Hui Jin, Tong-Zu Liu, Fang-Ming Chen, De-Gang Ding, Meng Fu, Xin-Quan Gu, Bang-Min Han, Xing Huang, Zhi Hou, Wan-Li Hu, Xin-Li Kang, Gong-Hui Li, Jian-Xing Li, Pei-Jun Li, Chao-Zhao Liang, Xiu-Heng Liu, Zhi-Yu Liu, Chun-Xiao Liu, Jiu-Min Liu, Guang-Heng Luo, Yi Luo, Wei-Jun Qin, Jian-Hong Qiu, Jian-Xin Qiu, Xue-Jun Shang, Ben-Kang Shi, Fa Sun, Guo-Xiang Tian, Ye Tian, Feng Wang, Feng Wang, Yin-Huai Wang, Yu-Jie Wang, Zhi-Ping Wang, Zhong Wang, Qiang Wei, Min-Hui Xiao, Wan-Hai Xu, Fa-Xian Yi, Chao-Yang Zhu, Qian-Yuan Zhuang, Li-Qun Zhou, Xiao-Feng Zou, Nian-Zeng Xing, Da-Lin He, Xing-Huan Wang, the Chinese Urological Doctor Association (CUDA), Urological Association of Chinese Research Hospital Association (CRHA-UA), Uro-Health Promotive Association of China International Exchange and Promotive Association for Medical and Health Care (CPAM-UHPA)

**Affiliations:** 1 Department of Urology, Institute of Urology, Zhongnan Hospital of Wuhan University, Wuhan, 430071 China; 2 Center for Evidence-Based and Translational Medicine, Zhongnan Hospital of Wuhan University, Wuhan, 430071 China; 3 Department of Urology, Tianjin Third Central Hospital Affiliated To Nankai University, Tianjin, 300170 China; 4 Department of Urology, Henan Provincial People’s Hospital, Zhengzhou University People’s Hospital, Zhengzhou, 450003 China; 5 Department of Urology, Beijing Tsinghua Changgung Hospital, School of Clinical Medicine, Tsinghua University, Beijing, 102218 China; 6 Department of Urology, China-Japan Union Hospital of Jilin University, Changchun, 130033 China; 7 Department of Urology, Shanghai General Hospital, Shanghai Jiao Tong University School of Medicine, Shanghai, 200080 China; 8 Department of Urology, Qinghai University Affiliated Hospital, Xi’ning, 810012 China; 9 Department of Urology, People’s Hospital of Hainan Province, Hainan Affiliated Hospital of Hainan Medical University Haikou, Haikou, 570311 China; 10 Department of Urology, Sir Run Run Shaw Hospital, Zhejiang University School of Medicine, Hangzhou, 310016 China; 11 Department of Urology, General Hospital of Ningxia Medical University, Yinchuan, 750003 China; 12 Department of Urology, The First Affiliated Hospital of Anhui Medical University, Hefei, 230022 China; 13 Department of Urology, Renmin Hospital of Wuhan University, Wuhan, 430060 China; 14 Department of Urology, The Second Hospital of Dalian Medical University, Dalian, 116023 Liaoning China; 15 Department of Urology, Zhujiang Hospital of Southern Medical University, Guangzhou, 510282 China; 16 Department of Urology, Guangdong Provincial People’s Hospital, Guangdong Academy of Medical Sciences, Guangzhou, 510080 China; 17 Department of Urology Surgery, Guizhou Province People’s Hospital, Guiyang, 550002 China; 18 Department of Urology, Xijing Hospital of Air Force Military Medical University, Xi’an, 710032 China; 19 Department of Urology, The 980St Hospital of the PLA Joint Logistics Support Force (Bethune International Peace Hospital of PLA), Shijiazhuang, 050082 China; 20 Department of Urology, Tangdu Hospital, The Air Force Military Medical University, Xi’an, 710038 China; 21 Department of Andrology, Jinling Hospital Affiliated to Nanjing University School of Medicine, Nanjing, 210002 China; 22 Department of Urology, Qilu Hospital of Shandong University, Jinan, 250012 China; 23 Department of Geriatrics, The Seventh Medical Center of Chinese, PLA General Hospital, Beijing, 100027 China; 24 Department of Urology, Beijing Friendship Hospital, Capital Medical University, Beijing, 100050 China; 25 Department of Urology, People’s Hospital of Tibet Autonomous Region, Lhasa, 850000 China; 26 Department of Urology, South China Hospital, Shenzhen University, Shenzhen, 518111 Guangdong China; 27 Department of Urology, The Second Xiangya Hospital of Central South University, Changsha, 410011 China; 28 Department of Urology, The First Affiliated Hospital of Xinjiang Medical University, Urumqi, 830054 China; 29 Department of Urology, Institute of Urology, Lanzhou University Second Hospital, Key Laboratory of Urological Diseases in Gansu Province, Lanzhou, 730030 China; 30 Department of Urology, Shanghai 9Th People’s Hospital, Shanghai JiaoTong University School of Medicine, Shanghai, 200011 China; 31 Department of Urology, Institute of Urology, West China Hospital, Sichuan University, 88 South Keyuan Road, Chengdu, 610041 China; 32 Department of Urology, The First People’s Hospital of Yunnan Province, Kunming University of Science and Technology, Kunming, 650041 China; 33 Department of Urology, The Fourth Hospital of Harbin Medical University, Heilongjiang Key Laboratory of Scientific Research in Urology, Harbin, 150001 China; 34 Department of Urology, The Affiliated Hospital of Inner Mongolia Medical University, Hohhot, 010059 China; 35 Department of Urology, Huaihe Hospital of Henan University, Kaifeng, 475000 Henan China; 36 Department of Urology, Tongji Hospital, Tongji Medical College, Huazhong University of Science and Technology, Wuhan, 430030 China; 37 Department of Urology, Peking University First Hospital, The Institute of Urology, Peking University, National Urological Cancer Center, Beijing, 100034 China; 38 Department of Urology, First Affiliated Hospital of Gannan Medical University, Ganzhou, 341000 Jiangxi China; 39 Department of Urology, National Cancer Center/National Clinical Research Center for Cancer/Cancer Hospital, Chinese Academy of Medical Sciences and Peking Union Medical College, Beijing, 100021 China; 40 Department of Urology, the First Affiliated Hospital of Xi’an Jiaotong University, Xi’an, 710061 China

**Keywords:** Transurethral plasmakinetic resection of prostate, Benign prostatic hyperplasia, Recommendation, Treatment, Guideline

## Abstract

Benign prostatic hyperplasia (BPH) is highly prevalent among older men, impacting on their quality of life, sexual function, and genitourinary health, and has become an important global burden of disease. Transurethral plasmakinetic resection of prostate (TUPKP) is one of the foremost surgical procedures for the treatment of BPH. It has become well established in clinical practice with good efficacy and safety. In 2018, we issued the guideline “2018 Standard Edition”. However much new direct evidence has now emerged and this may change some of previous recommendations. The time is ripe to develop new evidence-based guidelines, so we formed a working group of clinical experts and methodologists. The steering group members posed 31 questions relevant to the management of TUPKP for BPH covering the following areas: questions relevant to the perioperative period (preoperative, intraoperative, and postoperative) of TUPKP in the treatment of BPH, postoperative complications and the level of surgeons’ surgical skill. We searched the literature for direct evidence on the management of TUPKP for BPH, and assessed its certainty generated recommendations using the grade criteria by the European Association of Urology. Recommendations were either strong or weak, or in the form of an ungraded consensus-based statement. Finally, we issued 36 statements. Among them, 23 carried strong recommendations, and 13 carried weak recommendations for the stated procedure. They covered questions relevant to the aforementioned three areas. The preoperative period for TUPKP in the treatment of BPH included indications and contraindications for TUPKP, precautions for preoperative preparation in patients with renal impairment and urinary tract infection due to urinary retention, and preoperative prophylactic use of antibiotics. Questions relevant to the intraoperative period incorporated surgical operation techniques and prevention and management of bladder explosion. The application to different populations incorporating the efficacy and safety of TUPKP in the treatment of normal volume (< 80 ml) and large-volume (≥ 80 ml) BPH compared with transurethral urethral resection prostate, transurethral plasmakinetic enucleation of prostate and open prostatectomy; the efficacy and safety of TUPKP in high-risk populations and among people taking anticoagulant (antithrombotic) drugs. Questions relevant to the postoperative period incorporated the time and speed of flushing, the time indwelling catheters are needed, principles of postoperative therapeutic use of antibiotics, follow-up time and follow-up content. Questions related to complications incorporated types of complications and their incidence, postoperative leukocyturia, the treatment measures for the perforation and extravasation of the capsule, transurethral resection syndrome, postoperative bleeding, urinary catheter blockage, bladder spasm, overactive bladder, urinary incontinence, urethral stricture, rectal injury during surgery, postoperative erectile dysfunction and retrograde ejaculation. Final questions were related to surgeons’ skills when performing TUPKP for the treatment of BPH. We hope these recommendations can help support healthcare workers caring for patients having TUPKP for the treatment of BPH.

**Supplementary Information:**

The online version contains supplementary material available at 10.1186/s40779-022-00371-6.

## Background

Benign prostatic hyperplasia (BPH) is the most common benign disease leading to urination disorders in middle-aged and elderly men. The main histological manifestations include hyperplasia of prostatic stroma and glandular components, anatomically benign prostatic enlargement (BPE) leads to bladder outlet obstruction (BOO) and these urodynamics then cause lower urinary tract symptoms (LUTS) [1]. According to estimates from Global Burden of Diseases 2019 (GBD 2019), the number of BPH cases and the standardized incidence rate worldwide in 2019 were 11.26 million and 280.4/100,000 respectively, which indicates that BPH represents an important disease burden [2].

Transurethral resection of the prostate (TURP) is still the "gold standard" for minimally invasive surgical treatment of BPH using unipolar equipment [1, 3, 4]. The working electrode for TURP is located at the electrosurgical ring, and the loop electrode is located at the negative plate attached to the surface of the patient's body. The current from the working electrode forms a current loop through the patient's body, and non-ionic flushing solution (such as mannitol and glucose solution) is used. The hypotonic solution may be absorbed into the systemic circulation through the open prostate sinus, resulting in problems such as water intoxication leading to poor hemostatic effect or if serious, even to death. Thus, many minimally invasive procedures have emerged to replace the classic TURP. Bipolar TURP (B-TURP) is one such improvement. Both the working electrode and the loop electrode are located in the electrosurgical ring, the current does not need to pass through the patient's body, and the energy is limited between the working electrode and the loop electrode ("true bipolar" system) or the outer sheath ("quasi bipolar" system). This innovative method enables the resection to be carried out in isotonic electrolyte solution so producing its effect in conductive liquid by means of plasma, which theoretically avoids the occurrence of transurethral resection syndrome (TURS). The guideline concentrated on "true bipolar" system, and used "transurethral plasmakinetic resection of prostate (TUPKP)" to describe it to distinguish it from other systems.

In 2018, we published a guideline for the use of TUPKP for BPH in China [5]. Recently, a number of research papers are being published both in China and abroad providing research evidence for managing TUPKP that can change some of our previous recommendations and motivated us to update our guideline. This updated guideline includes three sections: the perioperative period of TUPKP in the treatment of BPH, postoperative complications of BPH and surgeon's surgical skill level.

## Methods

### Target users

General practitioners and urology nurses, geriatric, urology, teaching and research personnel engaged in TUPKP for the treatment of BPH.

### Target population

Patients with BPH requiring TUPKP surgery. The indications and contraindications of TUPKP surgery are detailed in Questions 1 and 2 as below.

### Composition of the guideline development group

Experts who were members of Project Groups for Minimally Invasive Plasma Surgery System of National Key Research and Development Program and Cloud Planning Solution, Professional Committee members of the Chinese Urological Doctor Association (CUDA), Urological Association of Chinese Research Hospital Association (CRHA-UA), Uro-Health Promotive Association of China International Exchange and Promotive Association for Medical and Health Care (CPAM-UHPA) composed the guideline steering committee, guideline development group and the guideline external review expert group.

The guideline panel was composed of a steering group, a working group, and an evidence search and synthesis group, which included 45 clinical experts (44 urological experts), 2 methodologists, and 16 clinical research assistants with evidence searching and assessment expertise. The external consultancy review group included 19 clinical experts and one methodologist. (See the Authors’ Contributions).

### Conflict of interest policy

All guideline panel members signed a confidentiality agreement and disclosed all potential conflicts of interest (Survey form see Additional file 1).

### Selection and identification of clinical questions and outcomes

The guideline development group formulated the selection forms for clinical questions and outcomes of “Transurethral Bipolar Plasmakinetic Prostatectomy Treatment for Benign Prostatic Hyperplasia in Chinese: Development of a National Evidence-based Clinical Practice Guideline” based on relevant published guidelines and systematic reviews, investigation of stakeholders, conference discussion, and expert consultation. After investigations and discussions, the guideline finally focused on three topics related to TUPKP in the treatment of BPH: the perioperative period relevant questions, postoperative complications and surgeon's surgical skill level and included 31 clinical questions with 21 outcomes. Outcomes were scored according to their importance from 1 to 9. The outcomes with a score greater than 7 were regarded as the critical outcomes.

Ordered by the importance determined by the expert score from high to low, the effective outcomes consisted of: maximum urinary flow rate (Q_max_) scores, postoperative quality of life (QoL), international prostate symptom score (IPSS), postoperative post-void residual (PVR), the International Index of Erectile Function-5 (IIEF-5), postoperative prostate volume (or intraoperative tissue weight), and prostate specific antigen (PSA); Safety outcomes consisted of: incidence of complications, urinary incontinence, intraoperative bleeding, urethral stricture, postoperative bleeding, operation time, thrombotic diseases (thrombus leads to catheter blockage or lower extremity deep venous thrombosis), capsule perforation, length of postoperative catheterization time, length of hospital stay, bladder flushing time, postoperative erectile dysfunction (ED), urinary tract infection (UTI) and retrograde ejaculation. Among them, the critical effective outcomes included postoperative Q_max_, QoL, IPSS and PVR. The critical safety outcomes consisted of the incidence of complications, urinary incontinence, intraoperative bleeding, urethral stricture and postoperative bleeding.

### Evidence review and development of clinical recommendations

We searched eight bibliographic databases (PubMed, Embase, Web of Science, The Cochrane Central Register of Controlled Trials, China National Knowledge Infrastructure, China Science and Technology Journal Database, Wanfang Data, Chinese BioMedical Literature Database) from inception to September 15, 2020. The methodologists designed search strategies (Additional file 2) using medical subject headings and text words in Chinese and English for all direct evidence, defined as systematic review or meta-analysis or original studies with no language limitation. In addition, we searched three representative guideline development professional societies (including European Association of Urology [EAU], American Urological Association [AUA], Canadian Urological Association [CUA], and six representative guideline databases (National Institute for Health and Clinical Excellence [NICE], Scottish Intercollegiate Guidelines Network [SIGN], World Health Organization [WHO], BMJ Best Practice, UpToDate, and YiMaiTong) and relevant urology monographs.

We first considered systematic reviews and meta-analysis published in professional medical journals. If there was no relevant systematic review and meta-analysis, we would consider formulating them based on existing primary research. If there was no relevant primary research, we would look for published guidelines, consensus, monographs and expert opinions.

The risk of bias and quality assessment was based on the international evaluation standards of the corresponding literature, ROB 2.0 for randomized controlled trials (RCT), ROBINS-I for non-randomized comparative intervention studies, and AMSTAR2 for systematic reviews and meta-analysis [6].

We developed the guidelines according to the WHO guideline development manual published in 2014. Meanwhile, we chose the grade criteria for evidence and recommendation used by the EAU (Table 1) [7]. Strong recommendations mean that most informed patients would choose the recommended management and that clinicians can structure their interactions with patients accordingly [8]. Weak recommendations mean that patients’ choices will vary according to their values and preferences, and clinicians must ensure that patients’ care is in keeping with their values and preferences [8]. We used the word “recommend” to introduce “strong recommendations”, and used “suggest” or “consider” to describe “weak recommendations”. The consensus principle of recommendation voting was as follows: if the number of votes of strength of a recommendation was more than 50%, the direction (such as support or oppose an intervention) and strength of recommendations can be determined directly; if the above standards cannot be met, but the total number of votes in the same direction of recommendation exceeds 70%, the direction of the recommendations can be determined, the strength of recommendations depends on the highest number of votes; if the above two items cannot be met, the next stage discussion shall be performed to reach an agreement.

**Table 1:** EAU Guideline’ levels of evidence and grades of recommendation

Level	Type of evidence
1a	Evidence obtained from meta-analysis of randomized trials
1b	Evidence obtained from at least one randomized trial
2a	Evidence obtained from one well-designed controlled study without randomization
2b	Evidence obtained from at least one other type of well-designed quasi-experimental study
3	Evidence obtained from well-designed non-experimental studies, such as comparative studies, correlation studies and case reports
4	Evidence obtained from expert committee reports or opinions or clinical experience of respected authorities

Grade	Nature of recommendations
Strong recommendations (for/against)	Advantages of interventions clearly outweigh the disadvantages or the disadvantages clearly outweigh the advantages
Weak recommendations (for/against)	Advantages and disadvantages of interventions are uncertain or the evidence regardless of its quality shows that the advantages and disadvantages are equal

The guidelines were reported in accordance with Appraisal of Guidelines for Research and Evaluation II (AGREE II) [6] and Reporting Items for Practice Guidelines in Healthcare (RIGHT) [9].

## Results

We finally used evidence from 12 original articles (included one RCT) and 9 systematic reviews or meta-analyses. We issued 36 statements. Among them, 23 were strong recommendations, and 13 were weak recommendations.

### Section 1. Perioperative (preoperative) relevant questions about TUPKP in the treatment of BPH

#### Question 1: What are the indications for TUPKP?

**Recommendation** TUPKP is one of the surgical methods used for BPH, and its indications are similar to others used for BPH surgery. TUPKP is recommended in the following situations: (1) BPH patients having moderate-to-severe LUTS (the IPSS is ranging from 8 to 35), which have significantly affected their quality of life, especially for those who have had poor results from drug treatment or refuse to receive drug treatment; (2) BPH patients with the following complications: (I) Recurrent urinary retention (cannot urinate at least once after catheter removal or two or more episodes of retention of urine); (II) Recurrent drug resistant hematuria; (III) Recurrent UTI; (IV) Bladder stones or diverticulum; (V) Secondary upper urinary tract hydronephrosis (with or without renal damage). (3) Patients having BPH combined with inguinal hernia, severe hemorrhoids or anal prolapse, where, in the clinician’s judgment, therapeutic effects cannot be achieved without removal of the lower urinary tract obstruction. **(Evidence Level: 4; Recommendation Strength Rating: Strong)**

**Evidence summary** We referred to the indications for surgical treatment of BPH in several Guidelines, such as EAU guidelines of management of non-neurogenic male LUTS (2020 version) [7], AUA Guideline on BPH/LUTS Surgical Treatment (2020 Edition) [10], CUA MLUTS/BPH guidelines (2018 version) [11], and *Guidelines for Diagnosis and Treatment of Urological and Andrological Diseases in China *(*2019 Version*) [12].

#### Question 2: What are the contraindications for TUPKP?

**Recommendation** The contraindications for TUPKP included: (1) Patients with severe cardiovascular and cerebrovascular diseases, respiratory diseases, hemorrhagic diseases, diabetes and liver and kidney dysfunction who cannot tolerate surgery; (2) Patients with severe UTI; (3) Patients with severe urethral stricture where after transurethral dilation the sheath of the resectoscope cannot pass through the stenosis; (4) There are congenital malformations of the spine and joints to such an extent that the lithotomy position cannot be adopted, which could affect the operator; (5) Neurogenic bladder. The above contraindications are not absolute. Under the premise of fully assessing the risk of surgery, most patients can still receive surgery if the conditions are suitable after adequate preparation. In addition, it is necessary to strengthen multidisciplinary cooperation and the training of practitioners. **(Evidence Level: 4; Recommendation Strength Rating: Strong)**

**Evidence summary** We referred to the transurethral bipolar plasmakinetic prostatectomy treatment for benign prostatic hyperplasia in Chinese: development of a national evidence-based clinical practice guideline (2018 Standard Edition) [5], Chinese experts’ consensus on the safety of transurethral plasmakinetic prostatectomy [13], book about prostate surgery [14] and the opinions of the expert group for these guidelines.

**Justification** The working electrode of TURP is located at the resection ring, and the return electrode is located on the negative plate attached to the patient's body. The electric current from the working electrode forms a current loop through the patient's body, which may cause malfunction of cardiac pacemakers. In contrast, the working electrode and the return electrode of the plasma bipolar resection are both located in the resection ring. The electric current does not pass through the patient's body, and the energy is limited to the area between the active electrode and the passive electrode. In theory, this can reduce the interference with the pacemaker. Therefore, wearing a pacemaker is not an absolute contraindication to TUPKP.

#### Question 3: What are the precautions for preoperative preparation of TUPKP in patients with renal impairment and UTI due to urinary retention?

**Recommendation** Preoperative preparation should follow the principles of surgery. (1) If the renal function is impaired by urinary retention, an indwelling catheter or suprapubic bladder puncture and fistula is recommended, and TUPKP should be performed after renal function improves. **(Evidence Level: 4; Recommendation Strength Rating: Strong)**; (2) If UTI is present, antimicrobial therapy is recommended prior to the operation until the infection is controlled. (**Evidence Level: 4**; **Recommendation Strength Rating: Strong)**

**Implementation consideration** We referred to Question 4 and Question 16 of this guideline for the use of antibacterial drugs.

**Evidence summary** We referred to the guideline for transurethral bipolar plasmakinetic prostatectomy treatment for benign prostatic hyperplasia in Chinese (2018 Standard Edition) [5] and the opinions of the expert group for these guidelines.

#### Question 4: Does preoperative prophylactic use of antibiotics reduce the risk of postoperative complications?

**Recommendation** (1) The prophylactic use of antibacterial drugs can reduce the risk of postoperative fever, bacteriuria, bacteremia and the use of additional antibacterial drugs. **(Evidence level: 4; Recommendation Strength Rating: Strong)**; (2) It is recommended that the prophylactic use of antimicrobial drugs follows the "Guiding Principles for the Clinical Application of Antimicrobial Drugs (2015 Edition)", and the choice and length of time antimicrobial drug are used should be determined based on the patient's condition. **(Evidence Level: 4; Recommendation Strength Rating: Strong)**

**Implementation consideration** Treatment of BPH by TUPKP is a clean-contaminated operation (type II incision), so this type of operation usually requires prophylactic antibacterial drugs. For the choice of perioperative prophylactic medication, the use of antibacterial drugs can be flexible in the absence of bacteriological results to guide the choice. Patients with negative urine cultures before surgery should use antibacterial drugs in accordance with the principles of clean-contaminated surgery. Fluoroquinolones or second-generation cephalosporins or broad-spectrum penicillins plus β-lactamase inhibitors are recommended. In view of the high resistance rate of Escherichia coli to fluoroquinolones in China, fluoroquinolones should be strictly controlled as prophylactic drugs during perioperative period. Considering the increase in bacterial resistance of antibacterial drugs, fosfomycin drugs can be used as alternatives based on current research. Choice of antibiotics for patients with asymptomatic bacteriuria before surgery should be based on sensitivity results to reduce the incidence of postoperative infective complications.

**Evidence summary** (1) A systematic review and meta-analysis including 10 placebo-controlled randomized controlled trials (RCTs) and 18 blank control RCTs (*n* = 4694) [15] evaluated the effect of prophylactic use of antibiotics for patients showing less than 100,000 colonies per milliliter of urine before the treatment of TURP. The results showed that: (I) The prophylactic use of antibiotics can significantly reduce the postoperative complications of TURP. The risk differences for post-TURP bacteriuria, high levels of fever, bacteremia and use of additional antibiotic treatment were − 0.17 (95% CI 0.20 to − 0.15), − 0.11 (95% CI − 0.15 to − 0.06), − 0.02 (95% CI − 0.04 to 0.00) and − 0.20 (95% CI − 0.28 to − 0.11), respectively. (II) There was no difference in postoperative catheterization or hospitalization time. (III) The adverse reactions are very few and mild, mainly including allergic reactions, fever and abdominal discomfort. Antibiotics recommended include natimicin, ceftriaxone sodium, enoxacin, amoxicillin, trimethoprim, cefoxitin, cefotaxime, aztreonam, cefnixi, pimecillin, menoxicillin, cefradine, cefuroxime, kanamycin, cephalosporin, cephalexin, ceftazidime, nitrofurantoin, cefoperazone, gentamicin, temoxicillin, and neonomine-trimethoprim. (2) A meta-analysis including 29 RCTs [16] (*n* = 4451) explored the efficacy and safety of fosfomycin tromethamine in the treatment of UTI. The results showed that: (I) In terms of clinical cure rate, it is equivalent to quinolones, β-lactams, furans and sulfonamides; (II) In terms of total clinical efficacy, it is comparable to quinolones, β-lactams, furans, aminoglycosides and sulfonamides. (III) In terms of bacterial clearance, it has similar effects to quinolones, β-lactams, furans, aminoglycosides and sulfonamides. (IV) In the incidence of adverse reactions, no difference was shown between fosfomycin and tromethamine when compared with quinolones, furans, aminoglycosides and sulfonamides, but was significantly lower than with β -lactam (RR = 0.56, 95% CI 0.34–0.94, *P* = 0.03).

We also referred to the Guiding Principles for the Clinical Application of Antimicrobial Drugs (2015 Edition) (Annex [2015] No. 43 of the National Health Office) [17], *Campbell-Walsh Urology* [18], and *Chinese Urology and Andrology Disease Diagnosis Treatment Guidelines *(*2019 edition*) [12].

**Justification** Currently, there are no RCTs, systematic reviews or meta-analyses dealing with preoperative use of antimicrobials to prevent postoperative infection in patients with BPH treated with TUPKP. One of the above pieces of evidence is the prophylactic use of TURP treatment, one is the result of fosfomycin treatment of UTI, and the others are recommended by the documents of national ministries and commissions in China and monographs.

### Section 2. Perioperative (intraoperative) relevant questions about TUPKP in the treatment of BPH

#### Section 2.1. Questions related to surgical operation

- **Question 5: What measures should be taken to reduce the risk of bladder explosion when intraoperative gas interferes with the surgical field in the treatment of BPH by TUPKP?**

**Recommendation** When TUPKP is used to treat BPH, attention should be paid to minimizing the entrance of external gas into the bladder. When air interferes with the surgical field of vision, it is suggested that as much as possible is removed. Other suggestions are; keeping the water circulation as smooth as possible during the operation, regularly draining and emptying it, and trying to avoid air accumulation and overfilling in the bladder. If the air cannot be expelled, try to avoid the electric cutting ring being excited by the air bubbles on the top anterior wall during the operation, and the inclination of the operating table can be changed to avoid air bubbles. **(Evidence Level: 4; Recommendation Strength Rating: Weak)**

**Implementation consideration** Expel the air in the lavage pipe before starting the electrical cut, replace the lavage fluid as soon as it is used up, and keep the lavage pipe tightly closed. The bladder should be emptied often during the operation. When the lavage fluid is discharged, the end of the resection lens can be slightly tilted to facilitate the discharge of air bubbles. After the negative pressure washer is full, the flushing operation can be carried out to reduce the air entering during flushing. Where large bubbles are seen, these should be emptied before continuing with the operation. Especially when using Ellick, pay attention to timely emptying.

**Evidence summary** We referred to the “Consensus on the Safety of Transurethral Plasma Resection of the Prostate” [13] and the opinions of the expert group of this guideline.

#### Section 2.2. Questions about adapting to the characteristics of different populations

- **Section 2.2.1. Efficacy and safety of TUPKP in the treatment of normal volume (< 80 ml) BPH**

- **Question 6: How does the efficacy and safety of TUPKP compare with TURP in the treatment of normal volume (< 80 ml) BPH?**

**Recommendation** Efficacy of TUPKP is equivalent to that of TURP in, and the safety is better. TUPKP is recommended. **(Evidence Level: 1a; Recommendation Strength rating: Strong)**

**Evidence summary** (1) A systematic review and meta-analysis [19] published in 2021 compared the efficacy and safety of TUPKP and TURP in the treatment of patients with normal volume (< 80 ml) BPH. A total of 42 RCTs with 6162 participants were included, including 3184 cases in the TUPKP group and 2978 cases in the TURP group. (I). Efficacy: In terms of the improvement of the IPSS, there was no statistical difference between TUPKP and TURP at the 6th, 12th and 60th months after surgery; In terms of the improvement of Q_max_ scores, there was no statistical difference between TUPKP and TURP at the 6th and 12th months after surgery, but TUPKP was better than TURP at the 60th month [weighted mean difference (WMD) = 1.55 ml/s, 95% CI (0.94–2.15)], the difference was statistically significant (*P* < 0.05); In terms of the IIEF-5, TUPKP was better than TURP at the 6th month after surgery (WMD = 4.80, 95% CI 3.79–5.81), the difference was statistically significant (*P* < 0.05), however, there was no statistical difference compared with TURP at the 12th and 60th months; In terms of PVR, TUPKP showed no statistical difference compared with TURP at the 6th and 12th month after surgery, but TUPKP was better than TURP at the 60th month (WMD = − 4.36 ml, 95% CI − 5.18 to − 3.54), the difference was statistically significant (*P* < 0.05); In terms of QoL, there was no statistical difference between TUPKP and TURP at 6, 12 and 60 months after surgery; and there was no statistical difference between TUPKP and TURP in the weight of the resected tissue. (II) Safety: TUPKP was better than TURP in operation time (WMD = − 5.15 min, 95% CI − 8.64 to − 1.65), hospitalization time (WMD = − 1.23 d, 95% CI − 1.80 to − 0.67), bladder irrigation time (WMD = − 1.23 d, 95% CI − 1.80 to − 0.67), intraoperative blood loss (WMD = − 45.16 ml, 95% CI − 79.07 to − 11.25), postoperative cauterization time (WMD = − 0.77 d, 95% CI − 1.04 to − 0.49) and postoperative urethral stricture (RR = 0.60, 95% CI 0.37–0.97), and the differences were statistically significant (*P* < 0.05); There was no statistical difference between the two methods in terms of capsule perforation, bladder spasm, urinary retention, dysuria, temporary urinary incontinence, UTI, ED, and retrograde ejaculation. (2) A network meta-analysis published in BMJ [20] (109 RCTs with a total of 13,676 subjects were included) found that in patients with prostate volume less than 60 ml, TUPKP was better than TURP in the terms of Q_max_ at the 12th month after surgery (WMD = 0.66 ml/s, 95% CI 0.03–1.30), and the difference was statistically significant (*P* < 0.05); the scores for Q_max_ at the 6th month and IPSS at the 6th and 12th month, showed no statistical difference between the two methods.

**Justification** Based on the above evidence, TUPKP has advantages over TURP and overcomes its shortcomings. TUPKP is equivalent to TURP in efficacy, but is better than TURP in terms of safety.

- **Question 7: How does the efficacy and safety of TUPKP compare with transurethral plasmakinetic enucleation of prostate (TUPEP) in the treatment of normal volume (< 80 ml) BPH?**

**Recommendation** For the treatment of normal volume (< 80 ml) BPH, both TUPKP and TUPEP are acceptable. It is suggested that clinicians consider practical issues based on their experience, the availability of equipment, and the patient's wishes. **(Evidence Level: 1a; Recommendation Strength Rating: Weak)**

**Evidence summary** A systematic review and meta-analysis published in 2021 [21] compared the efficacy and safety of TUPKP and TUPEP in the treatment of patients with normal volume (< 80 ml) BPH. A total of 24 RCTs with 2407 subjects were included, including 1202 cases in the TUPKP group and 1205 cases in the TUPEP group. (1) Efficacy: In the terms of IPSS score improvement, there was no statistical difference between TUPKP and TUPEP at the 6th and 12th months; in the terms of Q_max_, there was no statistical difference between TUPKP and TUPEP at the 6th and 12th month after surgery; In the terms of QoL, there was no statistical difference between the two surgery methods at the 6th and 12th months after surgery; in the terms of PVR, the TUPKP was worse than TUPEP at the sixth month (WMD = 1.54 ml, 95% CI 0.43–2.65) and the 12th month (WMD = 5.27 ml, 95% CI 0.57–9.97) after surgery, and the difference was statistically significant (*P* < 0.05); In the terms of IIEF-5, TUPKP was worse than TUPEP (WMD = − 0.81, 95% CI − 1.50 to − 0.12) at the 6th month after surgery, and the difference was statistically significant (*P* < 0.05); the amount of tissue removed in TUPKP was less than in TUPEP (WMD = − 8.54 g, 95% CI − 12.63 to − 4.46) and the difference was statistically significant (*P* < 0.05). (2) Safety: (I). TUPKP was worse than TUPEP in the operation time (WMD = 7.48 min, 95% CI 3.62–11.34), hospitalization time (WMD = 1.79 d, 95% CI 1.22–2.36), postoperative catheterization time (WMD = 0.97 d, 95% CI 0.69–1.25), bladder irrigation time (WMD = 11.07 h, 95% CI 9.01–13.13), capsule perforation (RR = 3.28, 95% CI 1.36–7.92), intraoperative blood loss (WMD = 51.88 ml, 95% CI 35.29–68.49) and bladder spasm (RR = 2.31, 95% CI 1.26–4.24), and these differences were statistically significant (*P* < 0.05); (II) There was no statistical difference in postoperative urethral stricture, temporary urinary incontinence, retrograde ejaculation, urinary retention, and UTI.

**Justification** Above evidence shows that, for patients with normal volume of BPH, the efficacy of TUPKP and TUPEP are generally equivalent, and TUPEP is better than TUPKP in terms of safety. However, in view of the low quality of the original studies included in this systematic review, which may affect the authenticity of the conclusions, it is suggested that clinicians consider practical issues based on their own experience, the availability of equipment, and the wishes of the patients. At the same time, large-scale, high-quality research studies are suggested to further demonstrate the reliability of these results.

- **Question 8: How does the efficacy and safety of TUPKP compare with open prostatectomy (OP) in the treatment of normal volume (< 80 ml) BPH?**

**Recommendation** Efficacy and safety of TUPKP is better than that of OP; because OP is more traumatic, TUPKP is recommended as the first choice. **(Evidence Level: 1b; Recommendation Strength Rating: Strong)**

**Evidence summary** There was one RCT [22] (*n* = 200) comparing the efficacy and safety of TUPKP and OP in the treatment of patients with normal volume (< 80 ml) BPH. (1) Efficacy: TUPKP was better than OP in the Q_max_, IPSS and QoL at the 6th month after surgery, and the difference was statistically significant (*P* < 0.05). (2) Safety: TUPKP was superior to OP in the terms of operation time, postoperative hospital stays, bladder irrigation time, cauterization time, ED, retrograde ejaculation, postoperative complications, and bladder spasm, and the differences were statistically significant (*P* < 0.05). At present, no relevant literature on the medium and long-term efficacy of TUPKP compared with OP surgery has been retrieved.

**Justification** The above evidence shows that the efficacy and safety of TUPKP are better than OP. The expert group that formulated this guideline believes that although OP can bring good therapeutic effect, it is not recommended because it is rarely used in clinical practice due to the large amount of trauma it generates and long postoperative recovery time.

- **Section 2.2.2. Efficacy and safety of TUPKP in the treatment of large-volume (≥ 80 ml) prostate**

- **Question 9: How does the efficacy and safety of TUPKP compare with TURP in the treatment of large-volume (≥ 80 ml) BPH?**

**Recommendation** In the treatment of large-volume BPH, TUPKP is as efficient as TURP, but is better than TURP in terms of safety. TUPKP is recommended as the first choice. **(Evidence Level: 1a; Recommendation Strength Rating: Strong)**

**Evidence summary** (1) A systematic review and meta-analysis published in 2021 [19] compared the efficacy and safety of TUPKP and TURP in the treatment of patients with large-volume (≥ 80 ml) BPH. Three RCTs with a total of 328 subjects were included, including 164 in the TUPKP group and 164 in the TURP group. (I). Efficacy: there was no statistical difference between the two methods in the terms of Q_max_ at 6–12 months after surgery; there was no statistical difference between the two methods in the terms of the weight of the resected tissue. (II) Safety: (i) TUPKP was better than TURP in operation time (WMD = − 7.20 min, 95% CI − 9.73 to − 4.67), hospitalization days (WMD = − 0.70 d, 95% CI − 1.12 to − 0.18) and intraoperative blood loss (WMD = − 140.84 ml, 95% CI − 179.62 to − 102.05), and the differences were statistically significant (*P* < 0.05); (ii) There was no statistical difference in catheterization time. (2) A network meta-analysis published in BMJ [20] (a total of 109 RCTs were included, *n* = 13,676) showed that in patients with prostate volume > 70 ml, there was no statistical difference between TUPKP and TURP at the 6th and the 12th month in the terms of Q_max_ and IPSS.

**Justification** The above evidence shows that TUPKP is as effective as TURP in the treatment of large-volume BPH, but is better than TURP in terms of safety. However, the current sample size is small and the included outcome indicators are few, and further research is needed to confirm these results.

- **Question 10: How does the efficacy and safety of TUPKP compare with TUPEP in the treatment of large-volume (≥ 80 ml) BPH?**

**Recommendation** For the treatment of large-volume (≥ 80 ml) BPH, both TUPKP and TUPEP are acceptable. Practical considerations should be made based on the experience of clinicians, the availability of equipment and the wishes of the patients. **(Evidence Level: 1a; Recommendation Strength Rating: Strong)**

**Evidence summary** A systematic review and meta-analysis published in 2021 [21] compared the efficacy and safety of TUPKP and TUPEP in the treatment of patients with large-volume (≥ 80 ml) BPH. A total of 7 RCTs with 811 subjects were included, including 424 cases in the TUPKP group and 387 cases in the TURP group. (1) Efficacy: there was no statistical difference between TUPKP and TUPEP in the QoL, Q_max_, PVR and IIEF-5 at the 6th month after surgery. TUPKP was worse than TUPEP in the terms of IPSS at the 6th month (WMD = 1.35, 95% CI 0.10–2.61), and was less than TUPEP in the excised tissue weight (WMD = − 12.85 g, 95% CI − 25.31 to − 0.38), and the differences were statistically significant (*P* < 0.05). (2) Safety: TUPKP was worse than TUPEP in the operation time (WMD = 15.50 min, 95% CI 2.40–28.60), bladder irrigation time (WMD = 33.56 h, 95% CI 5.25–61.86), postoperative catheterization time (WMD = 0.78 d, 95% CI 0.22–1.34), and length of stay (WMD = 0.89 d, 95% CI 0.23–1.55) with statistically significant difference (*P* < 0.05); There was no significant difference in the intraoperative blood loss, temporary urinary incontinence, postoperative urethral stricture, bladder spasm, capsule perforation, urinary retention or UTI.

**Justification** TUPKP and TUPEP are generally equivalent in the efficacy and safety in patients with volume of ≥ 80 ml. Based on the above evidence, TUPEP may be preferred by skilled physicians for patients with large BPH. However, the current sample size is limited and further clinical studies are needed to confirm it.

- **Question 11: How do the efficacy and safety of TUPKP compare with OP in the treatment of large-volume (≥ 80 ml) BPH?**

**Recommendation** For the treatment of large-volume (≥ 80 ml) BPH, the efficacy index of TUPKP is equivalent to that of OP, but the safety index is better than that of OP, so it is suggested that TUPKP is used. **(Evidence Level: 1a; Recommendation Strength rating: Weak)**

**Implementation consideration** The treatment of large-volume (≥ 80 ml) BPH with TUPKP is suggested by experienced physicians; Considering the difficulty of implementation in primary hospitals, OP can be used to treat large-volume (≥ 80 ml) BPH.

**Evidence summary** Guideline development team produced a systematic review and meta-analysis comparing the efficacy and safety of TUPKP and OP in patients with prostate volume ≥ 80 ml. A total of 2 RCTs [20, 21] were included. The total sample size was 214, including 108 cases in the TUPKP group and 106 cases in the OP group. The overall methodological quality of the included studies was low, and the two studies had a high risk of bias. Neither of the two studies reported the method of generating the random sequence, nor reported whether the random sequence was allocated or concealed; the two studies completed the surgical intervention according to the established surgical procedure and the risk was low; there was no missing data in the two studies and the risk was low; in the terms of the bias of outcome measurement, neither of the two studies reported blinding, and the risk was high; As for the bias of selective reporting, there was no research proposal in either of the studies, it was impossible to judge whether the results were reported selectively. (1) Efficacy: There was no statistical difference in IPSS, Q_max_, QoL and PVR at 6–12 months after surgery; there was no statistical difference in the weight of the resected tissue. (2) Safety: (I). TUPKP was better than OP in the intraoperative blood loss (WMD = − 69.20 ml, 95% CI − 95.01 to − 43.39), bladder flushing time (WMD = − 1.89 d, 95% CI − 3.66 to − 0.13), catheterization time (WMD = − 4.80 d, 95% CI − 5.22 to − 4.38), and hospitalization d (WMD = − 5.48 d, 95% CI − 6.54 to − 4.41), and the differences were statistically significant (*P* < 0.05); (II) The operation time for TUPKP was longer than OP (WMD = 40.40 min, 95% CI 34.66–46.15), and the difference was statistically significant (*P* < 0.05); (III) There was no statistical difference between the two surgery methods in postoperative UTI or urethral stricture.

Above evidence shows that the operation time for TUPKP in the treatment of large-volume BPH is longer than that of OP. TUPKP and OP are comparable in other efficacy and safety indexes. This guideline formulation expert group believes that although TUPKP has no advantage in operation time, it has advantages in terms of intraoperative blood loss, bladder irrigation time, catheter indwelling time, hospitalization days, and postoperative temporary urinary incontinence. Therefore, TUPKP is recommended. However, the current sample size is limited, and the included outcome indicators are few. There is still a lack of relevant health economics evidence for comparing the two methods. Further research is needed to verify the results.

- **Section 2.2.3. Efficacy and safety of TUPKP in the treatment of BPH in special populations**

- **Question 12: How do the efficacy and safety of TUPKP to treat high-risk populations [those having poor general health, with one or more diseased organs, especially heart, lung, liver, kidney disease, or the elderly (> 70 years)] compare with other surgical methods?**

**Recommendation** For high-risk patients with BPH, TUPKP is relatively safe. The multi-disciplinary team (MDT) recommended that experienced doctors conduct diagnosis and treatment to improve perioperative risk management. **(Evidence Level: 3; Recommendation Strength Rating: Strong)**

**Evidence summary** A systematic review and meta-analysis which included 18 case-series studies [23] (*n* = 1899) evaluated the efficacy and safety of TUPKP in the treatment of high-risk/advanced age patients with BPH. (1)Efficacy: The changes of Q_max_ at 1, 3, 6, 12 and 24 months after surgery were 12.28 ml/s (95% CI 8.42–16.14), 12.88 ml/s (95% CI 9.85–15.92), 14.32 ml/s (95% CI 10.47–18.18), 14.93 ml/s (95% CI 10.19–19.67) and 20.00 ml/s (95% CI 19.08–20.92), respectively; the changes of IPSS at 3, 6, 12, and 24 months were − 18.60 (95% CI − 23.20 to − 14.00), − 17.62 (95% CI − 20.21 to − 15.03), and − 19.14 (95% CI − 20.70 to − 17.59), − 19.06 (95% CI − 21.53 to − 16.60) and − 22.90 (95% CI − 24.26 to − 21.54), respectively; The changes of QoL at 1, 3, 6, 12 and 24 months after surgery were − 2.38 (95% CI − 4.26 to − 0.50), − 3.39 (95% CI − 4.57 to − 2.21), − 3.75 (95% CI − 4.14 to − 3.36), − 3.36 (95% CI − 4.56 to − 2.16) and − 4.58 (95% CI − 4.75 to − 4.41), respectively; the changes of PVR at 1, 3, 6, 12 and 24 months after surgery were − 231.16 ml (95% CI − 288.30 to − 174.01), − 76.10 ml (95% CI − 116.71 to − 35.50), − 159.90 ml (95% CI − 207.21 to − 112.59) and − 87.70 ml (95% CI − 91.91 to − 83.48), respectively. (2) Safety: (I). Compared with baseline data, the average operative time for TUPKP for the treatment of patients with high cardiovascular disease risk/elderly-aged was 49.62 min (95% CI 42.86–56.41), and the resected tissue weight was 42.29 g (95% CI 37.81–46.77), intraoperative blood loss was 76.28 ml (95% CI 57.48–95.07), hospital stay was 7.24 d (95% CI 5.24–9.23), catheterization time was 5.08 d (95% CI 3.42–6.75), postoperative serum sodium dropped to 2.00 mmol/l (95% CI 1.03–2.97), blood sugar dropped to 0.20 mmol/l (95% CI − 0.69 to 1.09), bladder flushing time was 29.76 h (95% CI 21.46–38.06); (II) The incidence of postoperative complications: urinary retention was 11% (95% CI 0–21%), temporary urinary incontinence was 6% (95% CI 5–7%), UTI was 9% (95% CI 7–13%), TURS was 1% (95% CI 0–2%), bleeding was 2% (95% CI 1–3%)), hematuria was 3% (95% CI 2–37%), temporary dysuria was 3% (95% CI 1–6%), urinary tract irritation was 15% (95% CI 11–22%), urethral stricture was 9% (95% CI 7–12%), perforation/false perforation was 4% (95% CI 1–11%), and death was 1% (95% CI 0–3%).

- **Question 13: Can TUPKP be used to treat people who take anticoagulant (antithrombotic) drugs? If so, should anticoagulant (antithrombotic) drugs be stopped before surgery?**

**Recommendation** For BPH patients taking anticoagulant (antithrombotic) drugs, when considering undertaking TUPKP, consultation with a cardiovascular physician and anesthesiologists before the operation is recommended to establish whether the drug should be stopped, bridging (dressing change), and postoperative resumption of medication, and the operation should be performed by an experienced urologist. (**Evidence Level: 3–4; Recommendation Strength Rating: Strong**).

**Evidence summary** A retrospective cohort study (*n* = 99) compared the safety and efficacy of TUPKP and TUPEP for patients on oral anticoagulants (OA) and/or platelet aggregation inhibitors (PAI) with benign prostatic obstruction (BPO) and having a gland size of > 60 g. Both groups demonstrated a significant improvement from baseline in terms of IPSS, QoL, Q_max_, and PVR volume values. Both procedures are safe and effective options in patients who are on OA and/or PAI [24]. A consensus mentioned that because TUPKP requires high visual field clarity, bleeding has a great impact on the safety of surgery. For patients with abnormal coagulation function, coagulation function should be evaluated in detail before operation and anticoagulant (antithrombotic) drugs or antiplatelet aggregation drugs should be stopped as appropriate [13].

Combined with the opinions of the expert group, it was believed that BPH patients taking anticoagulant (antithrombotic) drugs should receive surgery when they meet the surgical indications to improve their BPH symptoms and reduce the risk of hypertension and cerebral hemorrhage which is exacerbated by BPH.

**Justification** Currently, only one retrospective cohort study of patients with OA and/or PAI with large-volume BPH comparing TUPKP with TUPEP has been retrieved, and the evidence was limited. Although the current cardiovascular field has changed "anticoagulation" to "antithrombotic" and "dressing change" to "bridging", in order to facilitate the promotion of this guideline, we gave both the old and the new terminology.

### Section 3. Perioperative (postoperative) related questions about TUPKP treatment for patients with BPH

#### Section 3.1. Questions related to treatments after TUPKP

- **Question 14: What is the recommended time and speed of flushing after TUPKP?**

**Recommendation** It is suggested that normal saline flushing should be continued after TUPKP, and the length of time depends on the bleeding. Closely observe the color of the flushing fluid and stop flushing when the color turns clear. It is suggested that the flushing speed should be determined by the color of the flushing fluid. (**Evidence Level: 4; Recommendation Strength Rating: Weak)**

**Evidence summary** There is insufficient evidence; we referred to the guidelines for transurethral bipolar plasmakinetic prostatectomy treatment for benign prostatic hyperplasia in Chinese (2018 standard version) [5], Chinese experts’ consensus on the safety of transurethral plasmakinetic prostatectomy [13], the text book about prostate surgery [14] and the opinions of the expert group.

- **Question 15: How long should the indwelling catheter be left in place after TUPKP?**

**Recommendation** It is suggested that time of catheter removal should be considered holistically taking into account the patient's physical condition and the situation during the operation, generally it should be left in for no more than 3–5 d. (**Evidence Level: 4; Recommendation Strength Rating: Weak**).

**Evidence summary** There is insufficient evidence; we referred to the guidelines for transurethral bipolar plasmakinetic prostatectomy treatment for benign prostatic hyperplasia in Chinese (2018 standard version) [5], Chinese experts’ consensus on the safety of transurethral plasmakinetic prostatectomy [13] and the opinions of the expert group.

- **Question 16: What are the principles of postoperative therapeutic use of antibiotics?**

**Recommendation** The recommendation for therapeutic use of antibiotics is to follow the guidelines for clinical use of antibiotics (2015 Edition), and determine the selection and length of time of antibiotics use in accordance with the patient's condition. (**Evidence Level: 4; Recommendation Strength Rating: Strong**).

**Evidence summary** We referred to the guidelines for clinical use of antibiotics (2015 version) [17] jointly issued by the General Office of national health and Family Planning Commission, Office of the State Administration of Traditional Chinese Medicine and Pharmaceutical Equipment Bureau of the Ministry of Health of the General Logistics Department of the People's Liberation Army on July 24, 2015, and included the opinions of the expert group for this guideline.

#### Section 3.2. Questions related to follow-up after TUPKP treatment of BPH

- **Question 17: What is the recommended schedule and content of follow up appointments after TUPKP treatment of BPH?**

**Recommendation** It is recommended that after TUPKP patients have the first follow-up appointment 4–6 weeks after the catheter is removed to evaluate treatment response and assess any adverse events. The following tests are recommended: IPSS, uroflowmetry, PVR volume, routine urine test, and urinary tract ultrasound. Follow-up thereafter depends on the patient's condition. Urine culture can be performed when necessary. (**Evidence Level: 4; Recommendation Strength rating: Strong**).

**Implementation consideration** It should be noted that it is not guaranteed that patients will never get cancer after prostatectomy. Therefore, patients with long-term follow-up can decide whether to accept digital rectal examination and serum PSA determination if they so wish, so as to effect timely prostate cancer screening.

**Evidence summary** We referred to the EAU guidelines of management of non-neurogenic male LUTS (2020 version) [7], CUA MLUTS/BPH guidelines (2018 version) [11], *Guidelines for Diagnosis and Treatment of Urological and Andrological Diseases in China *(*2019 Version*) [12]and opinions of the expert group for this guideline.

### Section 4. Questions related to complications

#### Question 18: What are the types of complications and their incidence in the treatment of BPH with TUPKP?

**Recommendation** There are many types of complications in the treatment of BPH with TUPKP. Except for postoperative retrograde ejaculation and urinary tract irritation, the incidence of most complications is low, and the incidence of serious complications is extremely low, and the overall safety is good. **(Evidence Level: 3; Recommendation Strength Rating: Strong)**

**Evidence summary** A systematic review and meta-analysis published in 2021 [25] analyzed the types and incidence of complications of TUPKP in the treatment of BPH. A total of 27 case series reports were included, involving 5247 patients. One study was from Egypt and the others were from China. 17 studies reported the incidence of urethral stricture, 15 studies reported the incidence of transurethral resection syndrome (TURS), and 12 studies reported the incidence of temporary urinary incontinence, and the number of studies reporting other complications such as dysuria, the need for blood transfusion, postoperative bleeding was less than 10. The complication types and event rate (ER) when TUPKP was used in the treatment of BPH were as follows: retrograde ejaculation was 117/329 (ER = 24.77%, 95% CI 0.00–73.81%), urinary tract irritation symptom was 66/355 (ER = 17.15%, 95% CI 9.61–26.22%), TURS was 0/3102 (ER = 0, 95% CI 0–0%), acute urinary retention was 5 /132 (ER = 3.79%, 95% CI 1.08–7.85%), UTI was 40/1004 (ER = 3.43%, 95% CI 0.90%–7.21%), bladder spasm was 27/604 (ER = 6.94%, 95% CI 0.0–21.20%), urethral stricture was 135/4282 (ER = 3.37%, 95% CI 1.60–5.69%), temporary urinary incontinence was 71/1894 (ER = 3.73%, 95% CI 2.18–5.61%), postoperative blood transfusion was 46/2168 (ER = 2.94%, 95% CI 0.85–6.00%), BPH recurrence was 2/132 (ER = 1.52%, 95% CI 0.02–4.51%), capsular perforation was 23/550 (ER = 1.27%, 95% CI 0.00–5.94%), lower limb venous thrombosis was 1/182 (ER = 0.38%, 95% CI 0.00–2.20%), second operation was 32/2139 (ER = 0.98%, 95% CI 0.04–2.72%), obturator nerve reflex was 0/100 (ER = 0%, 95% CI 0–1.71%), epididymitis was 20/1065 (ER = 1.64%, 95% CI 0.10–4.58%), permanent urinary incontinence was 2/2913 (ER = 0%, 95% CI 0–0.01%), and ED was 15/401 (ER = 2.46%, 95% CI 0.09–6.90%).

**Justification** Based on the above evidence, the types of complications of TUPKP in the treatment of BPH are the same as those of other invasive interventions, but TUPKP has a higher level of safety. Among them, the highest incidence is retrograde ejaculation and urinary tract irritation, both of which are non-serious complications. The incidence of TURS, a serious complication, was zero.

#### Question 19: Can postoperative leukocyturia reflect the possibility of postoperative bacteriuria?

**Recommendation** Postoperative leukocyturia does not indicate postoperative bacteriuria. The possibility of postoperative bacteriuria cannot be judged only by postoperative leukocyturia. If the patient has no obvious infective symptoms, watchful waiting can be carried out; Urine culture is recommended if there are relevant infection symptoms. (**Evidence Level: 2b; Recommendation Strength Rating: Strong**).

**Evidence summary** A prospective self-controlled study (*n* = 121) prophylactically used ceftriaxone sodium in patients undergoing TUPKP, and collected two midstream urine samples from each patient after surgery, one for urine analysis (urinary leukocyte count), the other for urine culture. In 363 urine samples, the average concentration of leucocytes with and without bacteriuria was 323.9 and 297.6/μl (*P* > 0.05) respectively, that is, there was no significant correlation between bacteriuria and postoperative leukocyturia [26]. Bacterial culture is still the gold standard for diagnosing UTI.

**Justification** Leukopenia is a common symptom after TUPKP. Leukopenia may be related to the exudation of inflammatory cells from the prostate surgery wound. Leukopenia cannot reflect the possibility of postoperative bacteriuria. With the healing of the surgical wound, the leukopenia will normally improve naturally without clinical intervention.

#### Question 20: What are the treatment measures for perforation of the capsule and extravasation of fluid during the operation?

**Recommendation** Mild perforation of the capsule generally does not cause serious extravasation of the lavage fluid, and no special treatment is required. If a severe perforation occurs, a large amount of lavage fluid will enter the space around the bladder and the posterior peritoneal space, which will cause a large amount of lavage fluid to be absorbed. If it is found in time and there is not much exudate, the recommendation is to end the operation as soon as possible and administer postoperative diuretics, and it will generally resolve of its own accord; if there is much exudation and severe peritoneal irritation, suprapubic drainage is recommended. (**Evidence Level: 4; Recommendation Strength Rating: Strong**).

**Evidence summary** We referred to the guideline for transurethral bipolar plasmakinetic prostatectomy treatment for benign prostatic hyperplasia in Chinese [5], Chinese experts’ consensus on the safety of transurethral plasmakinetic prostatectomy [13] and the opinions of this guideline’s expert group.

**Justification** Capsule perforation and extravasation mainly occur during the operation, the main reason for which is that inexperienced practitioners can't recognize the prostate capsule clearly, and it is easy to excise too deeply, resulting in perforation of the capsule. Under high pressure, the flushing fluid can seep through the perforation site to the periphery of the prostate; if a severe capsular perforation occurs, a large amount of lavage fluid will enter the space around the bladder and the posterior peritoneal space, and be absorbed, which will cause the patient to experience symptoms such as abdominal distension, difficulty breathing, or heart failure. Therefore, it should be dealt with according to the degree of perforation and the time of discovery.

#### Question 21: What are the treatment measures for patients with TURS during the perioperative period?

**Recommendation** Close observation is recommended and the following measures should be taken: (1) Ensure smooth drainage to prevent the increase of bladder pressure due to poor drainage, which increases the absorption of irrigation fluid; (2) Monitor plasma electrolytes, central venous pressure, blood gas, urine volume, hematocrit, irrigation fluid absorption and cardiac conditions; (3) Diuretics may be used as appropriate for mild lavage fluid absorption; suprapubic and abdominal catheter drainage should be performed when there is much exudation and severe peritoneal irritation; For patients having nausea, vomiting, hypotension or hypertension, or disturbance of consciousness in the early postoperative period timely monitoring of electrolyte and plasma osmotic pressure is needed; (4) If necessary, please consult the ICU and cardiologist for treatment. (**Evidence Level: 4; Recommendation Strength Rating: Strong**).

**Evidence summary** We referred to the guideline for transurethral bipolar plasmakinetic prostatectomy treatment for benign prostatic hyperplasia in Chinese (2018 standard version) [5], Chinese experts’ consensus on the safety of transurethral plasmakinetic prostatectomy [13] and the opinions of this guideline’s expert group.

**Justification** TUPKP uses isotonic flushing solution and bipolar resection system so in theory this avoids the occurrence of TURS, but this type of situation may still be encountered in clinical practice. In fact, this is not caused by hyponatremia and water intoxication, but by the increase of circulating load, which can be life-threatening in severe cases.

#### Question 22: What are the treatment measures for postoperative bleeding?

**Recommendation** For mild bleeding, observe and temporarily speed up the flushing, confirm the catheter position, and pull the catheter to compress the electrosurgical wound; Severe bleeding should be stopped using an emergency resectoscope as soon as possible, and even open surgery can be considered to stop bleeding when necessary to ensure safety; For those with abnormal liver function or coagulation function, hemostatic drugs should be used as appropriate. Avoid premature activities after operation, increase anti infection measures and use stool softeners. The use of thrombin during perioperative period may have a preventive and therapeutic effect, but there is a risk of thrombosis, which needs to be comprehensively evaluated. (**Evidence Level: 3–4; Recommendation Strength Rating: Strong**).

**Evidence summary** A multicenter retrospective controlled study (*n* = 695) indicated that Hemocoagulase Bothrops Atrox can shorten the prothrombin time, hospitalization time and is probably safe among BPH patients undergoing TUPKP, exhibiting good hemostasis and coagulation efficacy, and would not be influenced by prostate volume [27]. High-quality and large sample studies, especially RCTs, are still needed. For specific treatment measures, we referred to the Transurethral bipolar plasmakinetic prostatectomy treatment for benign prostatic hyperplasia in Chinese: development of a national evidence-based clinical practice guideline (2018 standard version) [5] and the opinions of this guideline’s expert group.

#### Question 23: What are the treatment measures for patients with urinary catheter blockage after surgery?

**Recommendation** (1) Confirm whether the urinary catheter is in the normal position; (2) Check whether the excised tissue has been completely removed during the operation; (3) After the operation, the flushing speed should be adjusted according to the color of the flushing fluid to avoid the formation of blood clots; (4) If the catheter is blocked, use a syringe to pressurize and repeatedly suck out blood clots or tissue fragments as soon as possible until the obstruction is relieved; (5) Replace the urinary catheter. (**Evidence Level: 4; Recommendation Strength Rating: Strong).**

**Evidence summary** We referred to the guideline for transurethral bipolar plasmakinetic prostatectomy treatment for benign prostatic hyperplasia in Chinese (2018 standard version) [5], Chinese experts’ consensus on the safety of transurethral plasmakinetic prostatectomy [13] and the opinions of this guideline’s expert group.

#### Question 24: What are the treatment measures for postoperative bladder spasm?

**Recommendation** (1) (I). Eliminate the possibility of urinary catheter blockage. Adjust the flushing speed according to the color of drainage fluid to ensure timely drainage of small blood clots from the bladder; (II) Confirm whether the urinary catheter and airbag are in the normal position; (III) Active analgesia, spasmolysis and hemostasis: it is suggested that non-steroidal anti-inflammatory drugs are used for analgesia, along with opioids or patient-controlled epidural analgesia when necessary; Use anticholinergic drugs for spasmolysis; (IV) If necessary, diazepam can be administered orally for sedation. (**Evidence Level: 4; Recommendation Strength Rating: Weak**); (2) It is suggested that warm flushing fluid be used after the operation to reduce cold irritation to bladder. (**Evidence Level: 1a-2a; Recommendation Strength rating: Weak**).

**Evidence summary** A meta-analysis of RCT studies (*n* = 1665) showed that warming bladder irrigation fluid can reduce the occurrence of bladder spasm in patients after TURP (RR = 0.52, 95% CI 0.46–0.58) [28]; A non-randomized controlled study (*n* = 154) showed that maintaining the temperature of the bladder irrigation fluid at 30–35 ℃ can effectively reduce the occurrence of adverse reactions such as bladder spasm in patients after TUPKP [29]. Besides, we referred to *Campbell–Walsh Urology* [18] and the opinions of this guideline’s expert group.

**Justification** The meta-analysis was for the patients after TURP, which is indirect evidence; Above study for TUPKP was a non-randomized controlled study, lacking high-level evidence, so the recommendation level is weak.

#### Question 25: What are the treatment measures for postoperative overactive bladder?

**Recommendation** (1) Behavioral therapy, including changes in lifestyle, bladder training and pelvic floor muscle training, is the first-line treatment for bladder overactivity; (2) For patients having poor results from behavioral therapy, M receptor blockers, β_3_ receptor agonists or non-steroidal analgesics are suggested. (**Evidence Level: 4; Recommendation Strength Rating: Weak**).

**Evidence summary** We referred to the *Guidelines for Diagnosis and Treatment of Urological and Andrological Diseases in China *(*2019 Version*) [12], the guideline of transurethral bipolar plasmakinetic prostatectomy treatment for benign prostatic hyperplasia in Chinese (2018 standard version) [5], Chinese experts’ consensus on the safety of transurethral plasmakinetic prostatectomy [13] and the opinions this guideline’s expert group.

#### Question 26: What are the treatment measures for patients with urinary incontinence after surgery?

**Recommendation** (1) When handling the prostatic apical glands around the seminal caruncle during the operation, take care to protect the external urethral sphincter to avoid urinary incontinence caused by excessive resection; (2) After the diagnosis of postoperative urinary incontinence, non-surgical treatment is the first choice, including lifestyle adjustments (timed voiding, control of fluid intake, etc.), pelvic floor muscle function exercises, and oral medications. For urge urinary incontinence (UUI), or mixed urinary incontinence (MUI) dominated by UUI, the use of anticholinergic drugs or β_3_ receptor agonists is recommended for drug treatments; For stress urinary incontinence (SUI), there is no suitable drug recommended; (3) Surgical treatment is recommended for patients with no remission after 6–12 months of non-surgical treatment. For SUI, the recommendation is to offer a fixed sling or artificial urinary sphincter (AUS); for UUI, Onabotulinumtoxin A or sacral nerve stimulation is recommended, and consider bladder enlargement or urinary diversion if other schemes are ineffective. (**Evidence Level: 4; Recommendation Strength Rating: Strong**).

**Evidence summary** We referred to the EAU guideline of urinary incontinence (2020 version) [30], the *Guidelines for Diagnosis and Treatment of Urological and Andrological Diseases in China *(*2019 Version*) [12], the guideline for transurethral bipolar plasmakinetic prostatectomy treatment for benign prostatic hyperplasia in Chinese (2018 standard version) [5], Chinese experts’ consensus on the safety of transurethral plasmakinetic prostatectomy [13] and the opinions of this guideline’s expert group.

#### Question 27: What are the management measures for postoperative urethral stricture?

**Recommendation** Prevention of urethral stricture should be given priority. When faced with urethral stricture, urethral dilation, urethral stricture incision or urethral reconstruction is recommended according to the location and degree of the stricture. (**Evidence Level: 4; Recommendation Strength Rating: Strong**).

**Implementation consideration** Prevention of urethral stricture should be the main focus: (1) For patients with external urethral orifice stenosis before surgery, external urethrotomy can be performed during surgery; (2) Fully lubricate the cystoscope sheath; (3) The lens is advised to be inserted under direct vision during the operation; (4) Carefully choose properly sized surgical lens sheaths to avoid unnecessary trauma; (5) Postoperative indwelling catheters should not be too thick, and the indwelling time should not be too long; (6) Use minimal force when inserting and pulling out the catheter to prevent urethral mucosal damage caused by improper traction; (7) The appropriate timing for urinary catheter removal should be determined and catheter removal when the bladder is full can restore the patient's natural urination earlier and improve the success rate of natural urination.

**Evidence summary** We referred to the *Guidelines for Diagnosis and Treatment of Urological and Andrological Diseases in China *(*2019 Version*) [12], the guidelines for transurethral bipolar plasmakinetic prostatectomy treatment for benign prostatic hyperplasia in Chinese (2018 standard version) [5], Chinese experts’ consensus on the safety of transurethral plasmakinetic prostatectomy [13] and the opinions of this guideline’s expert group.

#### Question 28: What are the treatment measures for patients with rectal injury during surgery?

**Recommendation** Treat according to the principles of rectal injury. Rupture of the rectum should be repaired by laparotomy, and a sigmoid colostomy should be performed simultaneously, along with adequate drainage of the perirectal space to prevent the spread of infection. It is recommended that a colorectal surgeon is consulted for treatment. (**Evidence Level: 4; Recommendation Strength Rating: Strong**).

**Evidence summary** We referred to the guidelines for transurethral bipolar plasmakinetic prostatectomy treatment for benign prostatic hyperplasia in Chinese (2018 standard version) [5] and the opinions of this guideline’s expert group.

#### Question 29: What are the treatment measures for postoperative ED?

**Recommendation** ED is considered in relation to the patient's age, preoperative sexual function, degree of thermal injury and whether there has been prostate capsule perforation during operation. If ED occurs after operation, Phosphodiesterase type 5 (PDE5) inhibitor combined with psychological counseling can be considered for treatment. (**Evidence Level: 1b-4; Recommendation Strength Rating: Weak**).

**Evidence summary** One of the possible mechanisms of post-TURP ED is direct thermal injury to the erectile nerves, which run a few millimeters from the prostatic capsule. Other possible mechanisms are psychological effects of surgery and hospitalization, and cessation of sexual activity during the postoperative period [31]. A prospective observational study (*n* = 629) revealed that the only important factors associated with newly reported ED after TURP were diabetes mellitus (*P* = 0.003, *r* = 3.67) and observed intraoperative capsular perforation (*P* = 0.02, *r* = 1.12) [32]. This evidence is about ED after TURP, and so is indirect evidence.

EAU guidelines on ED, premature ejaculation, penile curvature and priapism (2019 version) [33], Andrology Branch of Chinese Medical Association guidelines for the diagnosis and treatment of ED (2016 version) [34] and the Chinese expert consensus on the use of tadalafil 5 mg once a day for the treatment of ED [35] showed that PDE5 inhibitor is currently the first-line drug for the treatment of ED. The commonly used clinical drugs include sildenafil, tadalafil and vardenafil, and treatment methods include "on-demand treatment" and "regular treatment". We also referred to Chinese experts’ consensus on the safety of transurethral plasmakinetic prostatectomy [13] and the opinions of this guideline’s expert group.

#### Question 30: What are the treatment measures for retrograde ejaculation after operation?

**Recommendation** Where patients need to manage retrograde ejaculation, sympathomimetic drugs are suggested, drug treatment with adrenergic receptor agonists may be successful in inducing antegrade ejaculation. If the drug treatment is ineffective or badly tolerated, and the patient has fertility issues, collection of sperm from the urine after orgasm for assisted reproduction is suggested. (**Evidence Level: 3–4; Recommendation Strength Rating: Weak**).

**Implementation consideration** (1) Patients need to be told before operation that retrograde ejaculation is a common complication of transurethral surgery, and the incidence is high after operation; (2) The retention of the bladder neck sphincter during operation may reduce the incidence of retrograde ejaculation.

**Evidence summary** A meta-analysis (*n* = 5247) of types and incidence of complications of TUPKP in the treatment of BPH showed retrograde ejaculation was the most common complication, 117 / 329 (ER = 24.77%, 95% CI 0.00–73.81%) [25]. A retrospective controlled study (*n* = 137) showed that TURP with bladder neck preservation can reduce the rate of retrograde ejaculation compared with standard TURP at the 3- month [n (%): 34 (58.6) vs. 69 (87.3), *P* < 0.001], 6- month [n (%): 19 (32.8) vs. 61 (77.2), *P* < 0.001] and 12-month [n (%): 19 (32.8) vs. 59 (74.7), *P* < 0.001] follow-ups [36]. Campbell-Walsh Urology mentioned that since the excision of bladder neck is part of the operation, ejaculation disorder becomes an important problem. Postoperative local hematoma formation, infection and fibrosis can cause sympathetic nerve damage, which will lead to the synergistic imbalance between the internal urethral sphincter and the external sphincter during ejaculation, leading to the occurrence of retrograde ejaculation [18]. The Chinese experts’ consensus on the safety of transurethral plasmakinetic prostatectomy mentioned that the reason for retrograde ejaculation may be that the normal structure of the bladder neck and internal urethral sphincter were damaged during the resection of the bladder neck gland, resulting in the dysfunction of the bladder neck which fails to close normally, resulting in the return of semen to the bladder during ejaculation. Therefore, the bladder neck sphincter should be preserved as far as possible to reduce the incidence of retrograde ejaculation [13].

For evidence on the treatment of retrograde ejaculation: A review on retrograde ejaculation showed medical and surgical strategies exist for the treatment of retrograde ejaculation. Medical strategies included Sympathomimetics drugs and adrenergic receptor agonists. Sympathomimetics stimulated the release of noradrenaline as well as activating alpha- and beta-adrenergic receptors, resulting in closure of the internal urethral sphincter, restoring antegrade flow of semen. But as time progressed their effect diminished. Surgical strategies included bladder neck reconstruction [37].

EAU guidelines on ejaculatory dysfunction (2004 version) mentioned that for retrograde ejaculation induction of antegrade ejaculation may be attempted using drug treatment. Imipramine, 25–75 mg 3 times a day; Ephedrine sulfate, 10–15 mg 4 times a day; Midodrin, 5 mg 3 times a day; Brompheniramine maleate, 8 mg twice a day; Or desipramine, 50 mg every second day are suggested. Alternatively, the patient can be encouraged to ejaculate when his bladder is full, to increase bladder neck closure. Sperm collection from the postorgasmic urine for use in assisted reproductive techniques is suggested if drug treatment is ineffective or not tolerated due to side-effects. When the patient has a spinal cord injury; drug therapy for inducing retrograde ejaculation cannot be used [38].

CUA Guideline: The workup and management of azoospermic males (2015 version) mentioned that since retrograde ejaculation may be due to the failure of the bladder neck to close at orgasm, the use of pseudoephedrine (60 mg before ejaculation) or similar alpha receptor agonists may close the bladder neck and convert retrograde ejaculation into antegrade ejaculation. If this is unsuccessful, sperm can usually be removed from the bladder (using a urine sample or catheter sample discharged after ejaculation) for assisted reproduction [39].

### Section 5. Questions related to the surgical skills of the surgeon undertaking TUPKP for the treatment of BPH

#### Question 31: Compared with other transurethral resections, what are the technical requirements for TUPKP in the treatment of BPH?

**Recommendation** (1) The basic operation techniques for TUPKP are the same as those for other transurethral resections. (**Evidence Level: 4; Recommendation Strength rating: Weak**); (2) Surgeons who are already proficient in TURP can consider directly performing TUPKP. (**Evidence Level: 4; Recommendation Strength Rating: Weak**); (3) Compared with TUPEP, the operational difficulty of TUPKP is lower. (**Evidence Level: 3; Recommendation Strength Rating: Weak**).

**Evidence summary** *Campbell-Walsh Urology* mentioned that TUPKP and TURP use almost identical methods for resection [18]. Three studies on the TUPEP learning curve of a single surgeon showed resection efficiency was stable after 40–55 cases [40–42]. For TUPKP, the learning curve reached a plateau after 20 cases [43], which indicated that TUPKP had a shorter learning curve than TUPEP. Combined with the opinions of the expert group, we believe that compared with TUPEP, the operational difficulty of TUPKP is lower.

## Discussion

During the development of this guideline, the research team systematically combed and summarized the literature evidence, adopted the standard guideline development methodology and completed the formulation of the guideline within a reasonable time. The following points should be noted for future research or future guideline updates: (1) Patient values/preferences are one of the three elements of evidence-based decision-making. The determination of recommendations also needs to refer to patients’ values or preferences. The formulation of this guideline didn’t conduct a survey of patients’ values/preferences. Next, the guideline development team will conduct a survey of preferences and values of patients who have undergone TUPKP in order to prepare for the update of this guideline. (2) The final determination of recommendations also requires appropriate reference to the relevant evidence of health economics [44, 45], but there is still a lack of health economics research on TUPKP itself and comparison of TUPKP with other surgical treatments in China. Therefore, it is necessary to carry out research in this area in the future. (3) Furthermore, there may be differences in physical fitness between Chinese, European and American populations. The adaptability of the patient’s body tissue to imported equipment and the comfort of the surgeon when using imported equipment also need to be studied. In addition, China currently advocates the independent research and development of medical equipment [46]. At present, there is still a lack of evidence for the comparison of efficacy and safety for TUPKP in the treatment of BPH when using domestic equipment versus imported equipment. Research in this direction also needs to be carried out in the future. (4) The current evidence from domestic and foreign populations suggests that the efficacy of TUPKP for the treatment of BPH is particularly related to the clinical experience of surgeons. The more experienced the surgeons, the better the effect of TUPKP treatments. Although China has been doing successful work in the training of doctors' skills, there is still a lack of relevant research on the training of doctors in China. (5) For the high-risk population, there is a lack of research on the comparison between TUPKP and other surgical treatments; there is still a lack of relevant research on people taking anticoagulants, which also needs work to be carried out in the future. (6) There is a lack of relevant research on details of nursing issues. In the future, it may be necessary to answer nursing related questions with the help of real-world evidence. (7) As shown in the evidence summary in this guideline, many questions lack research evidence, and some questions lack high-quality research evidence. These areas urgently need high quality clinical trials to provide the needed evidence, especially localization evidence.

## Supplementary Information

**Additional file 1.** Conflict of interest statement form.

**Additional file 2.** Literature retrieval strategy.

## Data Availability

All data generated or analyzed during this study are included in this published article and its supplementary information files.

## Abbreviations

AUS: Artificial urinary sphincter
AUA: American Urological Association
BOO: Bladder outlet obstruction
BPE: Benign prostatic enlargement
BPH: Benign prostatic hyperplasia
BPO: Benign prostatic obstruction
CUA: Canadian Urological Association
EAU: European Association of Urology
ED: Erectile dysfunction
ER: Event rate
ICU: Intensive care unit
IIEF-5: International Index of Erectile Function-5
IPSS: International prostate symptom score
LUTS: Lower urinary tract symptoms
MDT: Multi-disciplinary team
MUI: Mixed incontinence
NICE: National Institute for Health and Clinical Excellence
OA: Oral anticoagulants
OP: Open prostatectomy
PAI: Platelet aggregation inhibitors
PSA: Prostate specific antigen
PVR: Post-void residual
Q_max_: Maximum flow rate
QoL: Quality of life
RCTs: Randomized Controlled Trials
SIGN: Scottish Intercollegiate Guidelines Network
SUI: Stress urinary incontinence
TUPEP: Transurethral plasmakinetic enucleation of prostate
TUPKP: Transurethral plasmakinetic resection of prostate
TURP: Trans urethral resection of the prostate
TURS: Transurethral resection of prostate syndrome/transurethral resection syndrome
UI: Urinary incontinence
UTI: Urinary tract infection
UUI: Urge urinary incontinence
WMD: Weighted mean difference
