# Supplementary material for: Clinical practice guideline for transurethral plasmakinetic resection of prostate for benign prostatic hyperplasia (2021 Edition)
Source: Mil Med Res. 2022 Apr 1;9:14. doi: 10.1186/s40779-022-00371-6 (PMC8974007; doi:10.1186/s40779-022-00371-6)
Supplement: Supplementary file 1 — Additional file 1. Conflict of interest statement form. [file 40779_2022_371_MOESM1_ESM.docx]

**Conflict of interest statement form**

All guidelines steering committee, guideline development team (including consensus expert group and evidence synthesis and translation working group) and guidelines external review team must disclose all potential conflicts of interest (e.g. all interests that affect or may affect the objectivity and independence of the experts).

On this conflict of interest statement, you must disclose any commercial, professional or other conflicts of interest related to the subject of this guideline, and any interests that may be affected by the results of this guideline.

| Name |  | Sex |  |
| --- | --- | --- | --- |

**1. Declaration of economic conflict of interest**

(Note: 1. The companies that are interested in this guideline mentioned in the following questions are those that develop, produce, sell, and promote various medical devices and equipment related to transurethral plasmakinetic resection of prostate, monopolar resection, enucleation and open prostatectomy for the treatment of benign prostatic hyperplasia (BPH), and those related to perioperative management of drugs, preparations, medical devices or equipment, etc. 2. Immediate family members, refer to people who have a direct blood relationship or marriage relationship with oneself, including spouses, parents, and children.)

| **Within the past** 3 years**:** | |
| --- | --- |
| I or my immediate family members hold stocks in companies that have an interest in the guideline | YES□ NO□ |
| I or my immediate family members have been invited to serve as consultants for companies interested in this guideline and receive corresponding remuneration | YES□ NO□ |
| I or my immediate family members have received fees from companies interested in this guideline to support research | YES□ NO□ |
| I or my immediate family members have accepted other expenses (such as travel expenses) of more than 10,000 from a company that has an interest in this guideline | YES□ NO□ |

**2. Statement of non-economic conflicts of interest**

| **Within the past** 3 years**:** | |
| --- | --- |
| I have published a paper comparing transurethral plasmakinetic resection of prostate and monopolar resection for the treatment of BPH | YES□ NO□ |
| I have published a paper comparing transurethral plasmakinetic resection of prostate and enucleation for the treatment of BPH | YES□ NO□ |
| I have published a paper comparing transurethral plasmakinetic resection of prostate and open prostatectomy for the treatment of BPH | YES□ NO□ |
| I have published papers related to the perioperative management of transurethral plasmakinetic resection of prostate for the treatment of BPH | YES□ NO□ |
| I have published papers related to the complications of transurethral plasmakinetic resection of prostate for the treatment of BPH | YES□ NO□ |
| I have applied for a patent related to transurethral plasmakinetic resection of prostate for the treatment of BPH | YES□ NO□ |
| I have applied for a patent related to transurethral monopolar resection for the treatment of BPH | YES□ NO□ |
| I have applied for a patent related to transurethral plasmakinetic enucleation of prostate for the treatment of BPH | YES□ NO□ |
| I have applied for a patent related to open prostatectomy for the treatment of BPH | YES□ NO□ |
| I have applied for a patent related to the perioperative management of transurethral plasmakinetic resection of prostate for the treatment of BPH | YES□ NO□ |

In addition to the above, in the past 3 years, what else do you need to declare? Yes □ No □

If your answer to any of the above questions is "yes", please give a brief explanation in the box below.

|  |
| --- |

**Informed about the conflict of interest statement**: I agree to make the above completed content public to other guideline development members, and agree that the statement of interest form will be published in the guideline.

**Statement**: I promise that what I have stated is true and complete. If the information I have stated above changes at any time, I will promptly inform the guideline secretary group and complete a new statement of interest form.

**Signed:**  **Date:**
