# Supplementary material for: Clinical practice guideline for transurethral plasmakinetic resection of prostate for benign prostatic hyperplasia (2021 Edition)
Source: Mil Med Res. 2022 Apr 1;9:14. doi: 10.1186/s40779-022-00371-6 (PMC8974007; doi:10.1186/s40779-022-00371-6)
Supplement: Supplementary file 2 — Additional file 2. Literature retrieval strategy. [file 40779_2022_371_MOESM2_ESM.docx]

**Literature retrieval strategy.** Taking PubMed as an example, the literature retrieval strategy was shown below.

#1 Prostatic Hyperplasia [Mesh] OR “Prostatic Hyperplasia”[All Fields] OR “Prostatic Hypertrophy” [All Fields] OR “Benign Prostatic Hyperplasia” [All Fields] OR lower urinary tract symptoms [Mesh] OR “lower urinary tract symptom*” [All Fields] OR LUTS [All Fields] OR “lower urinary tract obstruction” [All Fields] OR “bladder outlet obstruction” [All Fields] OR BOO[All Fields] OR “Bladder Neck Obstruction” [All Fields] OR “Urinary Bladder Neck Obstruction” [All Fields] OR Urinary Bladder Neck Obstruction [Mesh] OR “Urinary Bladder Neck Obstruction” [All Fields] OR urine retention [Mesh] OR “urine retention” [All Fields] OR “urinary retention” [All Fields] OR uroschesis [All Fields] OR “acute urinary retention” [All Fields] OR “chronic urine retention” [All Fields] OR “chronic urinary retention” [All Fields] OR “transurethral resection of the prostate syndrome*” [All Fields] OR “bladder stone*” [All Fields] OR “bladder infection” [All Fields] OR “secondary upper urinary tract hydrop*” [All Fields] OR “upper urinary tract infection” [All Fields]

#2 “transurethral bipolar plasmakinetic prostatectomy” [All Fields] OR “transurethral plasma bipolar electrotomy” [All Fields] OR “transurethral plasmakinetic prostatectomy” [All Fields] OR “transurethral plasmakinetic resection of prostate” [All Fields] OR “transurethral plasmakinetic resection of the prostate” [All Fields] OR “transurethralplasma kinetic resection of the prostate” [All Fields] OR “plasma kinetic resection of the prostate” [All Fields] OR “bipolar plasmakinetic transurethral resection of the prostate” [All Fields] OR “bipolar transurethral resection of the prostate” [All Fields] OR “bipolar plasmakinetic transurethral resection of prostate” [All Fields] OR “endoscopic bipolar prostate resection” [All Fields] OR TUPKP [All Fields] OR PKRP [All Fields] OR TUPKRP [All Fields] OR BP-TURP [All Fields] OR TKRP [All Fields] OR B-TURP [All Fields] OR “Bipolar TURP” [All Fields] OR TUR-PK [All Fields] OR TUPKVP [All Fields] OR PKVP [All Fields] OR TUP-KVP [All Fields] OR “transurethral electrovaporization of the prostate” [All Fields] OR TUVP [All Fields]

#3 “envelope perforation” [All Fields] OR “capsule perforation” [All Fields] OR “envelope extravasation” [All Fields] OR “capsule extravasation” [All Fields] OR “transurethral resection syndrome*” [All Fields] OR TURS [All Fields] OR “rectal injur*” [All Fields] OR “postoperative bleeding” [All Fields] OR “secondary hemorrhage” [All Fields] OR “intraoperative and postoperative bleeding” [All Fields] OR “hematuria” [All Fields] OR “haematuria” [All Fields] OR “hematuresis” [All Fields] OR “blocked catheter” [All Fields] OR “postoperative bladder spasm” [All Fields] OR “postoperative lower urinary tract symptom*” [All Fields] OR LUTS [All Fields] OR “postoperative LUTS” [All Fields] OR “postoperative overactive bladder” [All Fields] OR “postoperative OAB” [All Fields] OR “postoperative urinary incontinence” [All Fields] OR “postoperative urethral stricture” [All Fields] OR “postoperative recurrence” [All Fields] OR “urinary irritation” [All Fields] OR “urinal tract infection after urethral catheterization” [All Fields] OR epididymitis [All Fields] OR “Postoperative urinary retention” [All Fields] OR “Postoperative bacteriuria” [All Fields] OR post-bacteriuria [All Fields] OR “provisionality incontinence” [All Fields] OR “Temporary incontinence” [All Fields] OR “temporary urinary incontinence” [All Fields] OR “permanency urinary incontinence” [All Fields] OR “permanent incontinence” [All Fields] OR “clot retention” [All Fields] OR “sexual dysfunction” [All Fields] OR “sex disorder*” [All Fields] OR “sexual disturbance” [All Fields] OR “sexuality disfunction” [All Fields] OR “postoperative erectile dysfunction” [All Fields] OR  ”erectile dysfunction” [All Fields] OR “ED” [All Fields] OR “retrograde ejaculation” [All Fields] OR “libido disorder” [All Fields] OR “sexual dysfunction” [All Fields] OR “coitus disorder” [All Fields] OR “ejaculation disorder” [All Fields] OR “ejaculation disorder” [All Fields] OR “defective ejaculation” [All Fields]

#4 #1 AND #2

#5 #2 AND #3

#6 #4 OR #5
